# Effective Drugs Against Severe Fever With Thrombocytopenia Syndrome Virus in an *in vitro* Model

**DOI:** 10.3389/fmed.2022.839215

**Published:** 2022-03-31

**Authors:** Mi-Seon Bang, Choon-Mee Kim, Dong-Min Kim, Na Ra Yun

**Affiliations:** ^1^Department of Internal Medicine, College of Medicine, Chosun University, Gwangju, South Korea; ^2^Premedical Science, College of Medicine, Chosun University, Gwangju, South Korea

**Keywords:** antiviral activity, peramivir, nitazoxanide, favipiravir, severe fever with thrombocytopenia syndrome virus (SFTSV)

## Abstract

**Background:**

Severe fever with thrombocytopenia syndrome (SFTS) is an emerging tick-borne infectious disease caused by the SFTS virus (SFTSV). This syndrome is endemic in China, South Korea, and Japan, with a fatality rate of approximately 20–30%. Although the World Health Organization has listed SFTS as a disease that requires urgent steps for the development of its treatment, no treatments are available.

**Methods:**

We analyzed the antiviral activity of 41 drugs against the SFTSV to explore potential therapeutic candidates using real-time reverse transcription-polymerase chain reaction and plaque assay *in vitro*.

**Results:**

Peramivir, nitazoxanide, and favipiravir were found to have inhibitory effects on the SFTSV at concentrations below the maximum plasma concentration (Cmax). The concentrations that inhibited the SFTSV by 50% were as follows: peramivir, half maximal effective concentration (EC_50_) 12.9 μg/mL; nitazoxanide, EC_50_ 0.57 μg/mL; and favipiravir, EC_50_ 4.14 μg/mL.

**Conclusion:**

The effects of peramivir and nitazoxanide on the SFTSV were identified for the first time in this study. Future studies need to include animal models of SFTSV infection, clinical trials including dose-ranging trials, and evaluation of combination therapy with other potential antivirals.

## Introduction

Severe fever with thrombocytopenia syndrome (SFTS) was first reported in China in 2011 as an emerging tick-borne infectious disease (1). The causative agent—the SFTS virus (SFTSV)—was subsequently identified in South Korea and Japan in 2013, following lethal infections in humans (2, 3). The SFTSV has been classified into the genus *Banyangvirus* of the family Phenuiviridae (1). SFTS is endemic in China, South Korea and Japan (2, 3), with a fatality rate of approximately 20–30% (4, 5). In addition, SFTS cases endemic to Vietnam (6), Taiwan (7), and Xinjiang in China have been reported (8), indicating that the distribution of the SFTSV in Southeast Asia might be an underestimation (9).

The SFTSV is a negative-stranded RNA virus, and its genome comprises three segments: large (L), medium (M), and small (S) that encode the RNA-dependent RNA polymerase (RdRp), glycoprotein precursors (Gn and Gc), and nucleoprotein and non-structural proteins, respectively (1). The RdRp is responsible for viral replication and is a major target of nucleoside analogs, which are used as therapeutic antiviral drugs (1).

Although the World Health Organization lists SFTS as a disease requiring immediate attention, there are no effective treatments (9, 10). Recent studies have identified effective antiviral agents against the SFTSV, both *in vitro* and *in vivo*, with favipiravir and ribavirin being the most promising candidates (11, 12).

In this study, we analyzed the activity of 41 antiviral agents against the SFTSV to explore their therapeutic potential for the treatment of SFTS using real-time reverse transcription-polymerase chain reaction (RT-PCR) and plaque assay, *in vitro*.

## Materials and Methods

### Viral and Cell Culture

In this study, we used a clinically isolated SFTSV strain, KADGH/2013/Korea (GenBank accession no. KU507553), which was propagated in Vero E6 cells. The viral titer was determined on the basis of the viral RNA copy number after 10-fold serial dilution, using real-time RT-PCR. All infection experiments were performed in a biosafety level-3 laboratory of the Health and Environment Research Institute of Gwangju City.

The African green monkey kidney Vero E6 cell line, used in this study, was purchased from the Korean Cell Line Bank (KCLB no. 21587) and maintained in Dulbecco’s modified Eagle’s medium (DMEM, Gibco, Thermo Fisher Scientific, United States) supplemented with 10% fetal bovine serum (FBS, Gibco) at 37°C in a humidified atmosphere with 5% CO_2_.

### Drugs

Acetaminophen (catalog number HY-66005), arbidol hydrochloride (HY-14904A), aspirin (HY-14654), atazanavir sulfate (HY-17367A), baloxavir marboxil (HY-109025), daclatasvir (HY-10466), darunavir (HY-17040), dolutegravir (HY-13238), efavirenz (HY-10572), elbasvir (HY-15789), etravirine (HY-90005), galidesivir (BCX4430, HY-18649A), ganciclovir (HY-13637), glecaprevir (HY-17634), hydroxychloroquine (HY-B1370), indomethacin (HY-14397), ledipasvir (HY-15602), lopinavir (HY-14588), losartan (HY-17512), nafamostat mesylate (HY-B0190A), nitazoxanide (HY-B0217), ombitasvir (HY-13997), oseltamivir (HY-13318), penciclovir (HY-17424), perindopril salt (HY-B0130A), raltegravir (HY-10353), remdesivir (GS-5734, HY-104077), rilpivirine (HY-10574), ritonavir (HY-90001), simeprevir (HY-10241), sofosbuvir (HY-15005), and β-d-N4-hydroxycytidine (HY-125033) were purchased from MedChemExpress (Monmouth Junction, NJ, United States). Amodiaquin dihydrochloride dihydrate (A2799), artesunate (A3731), chloroquine phosphate (PHR1258), mefloquine hydrochloride (PHR1705), peramivir trihydrate (SML2486), ribavirin (R9644), teicoplanin (T0578), and toremifene citrate salt (T7204) were purchased from Sigma-Aldrich (St, Louis, Missouri, United States). Favipiravir (T-705) was provided by the Toyama Chemical Co., Ltd. (Japan). The drug solutions were filtered through a 0.22 μM membrane and were stored at −80°C.

### Antiviral Activity

To evaluate the antiviral efficacy of the drugs, Vero E6 cells were cultured in 24-well cell culture plates at a density of 2.5 × 10^5^ cells per well for 24 h. The cells were washed with sterile phosphate-buffered saline (PBS), and 5 × 10^5^ viral RNA copies, i.e., approximately 100 plaque forming units (PFUs), were added per well, and the plate was incubated for 1 h. The virus was removed, and the cells were washed thrice with PBS. The cells were then treated with maximum plasma concentration (Cmax) doses of the antiviral drugs in DMEM with 10% FBS for 48 h. The supernatant was collected for further quantifications.

### Viral RNA Extraction and Real-Time Reverse Transcription-Polymerase Chain Reaction

Viral RNA was extracted from 200 μL of the supernatant of the infected cells using a viral DNA/RNA extraction kit (ZiXpress, Cat no. ZP02201) in an automated nucleic acid purification system (ZiXpress-32) according to the manufacturer’s instructions. The RNA was eluted in 100 μL of RNase-free water. cDNA was synthesized using a SuperScript^®^ VILO™ MasterMix (catalog number 11755, Invitrogen, Thermo Fisher Scientific, United States) the following conditions: 50°C for 30 min, 95°C for 10 min. The SFTSV was detected using the Bioneer Exicycler 96 Real-time PCR kit (Bioneer Inc., Korea). The nucleocapsid protein gene of the S segment of the SFTSV was amplified by real-time RT-PCR from the RNA template using the following primers: SQ-F (5′-ACCTCTTTGACCCTGAGTTWGACA-3′), SQ-R (5′-CTGAAGGAGACAGGTGGAGATGA-3′) and probe (5′-[FAM] TGCCTTGACGATCTTA [BHQ1]-3′) (13). A standard curve was generated by determining the copy numbers from serial dilutions (10^1^–10^8^ copies) of the target gene cloned plasmid. PCR was performed using the following conditions: 45 cycles at 95°C for 15 s and 60°C for 45 s.

### Plaque Assay

To explore the antiviral efficacy of the drugs, Vero E6 cells were cultured in 24-well cell culture plates at a density of 2.5 × 10^5^ cells per well for 24 h; They were washed with PBS, and SFTSV of 50–100 PFUs per well were added to the cells for 1 h. The virus was removed and washed three times with PBS. The cells were then treated with 2-fold serial dilutions of Cmax doses of the antiviral drugs in DMEM with 5% FBS and 1% methyl cellulose. At the end of the 10-day incubation period, the overlay medium was fixed by adding 1 mL of acetone:methanol (1:1) solution. Crystal violet (1%) solution was added to each well and allowed to stain for 20–30 min. The cell monolayer in each well was rinsed with approximately 1 mL of water, and the plates were allowed to air dry, and the viral plaques were counted, subsequently.

### Data Analysis

The results obtained from the genome quantification and plaque assays at 50% effective concentration (EC_50_) of each drug were analyzed using GraphPad Prism 8.0.1 (GraphPad software, San Diego, CA, United States).

## Results

### Screening for Effective Drugs With Antiviral Activity Against the Severe Fever With Thrombocytopenia Syndrome Virus

**FIGURE 1:**
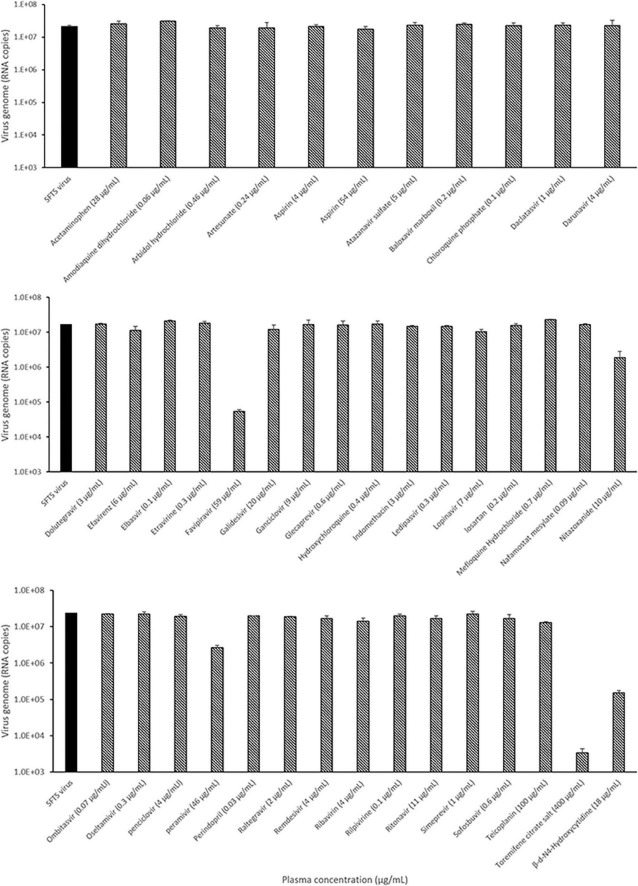
Identification of the candidate drugs inhibiting SFTSV replication using real-time RT-PCR. Graphs depict mean RNA copies of the SFTSV genome (left y-axis) upon treatment with the plasma concentration (Cmax) dose of the drug. Vero E6 cells were infected with 5 × 10^5^ RNA copies of SFTSV at the Cmax dose of the drug for 48 h, after which replication was determined through quantitation of the S segment of the SFTSV genome. Only SFTSV (black bar); SFTSV with drug treatment (black and white bar).

The antiviral activity of 41 antiviral drugs was measured at the Cmax to screen drugs that suppress SFTSV replication by more than 50%. The SFTSV RNA present in the supernatant was quantified using real-time RT-PCR after evaluating the Cmax of each drug in Vero E6 cells infected with the SFTSV for 48 h. Quantification analysis revealed that 5 of the 41 drugs, namely, favipiravir, nitazoxanide, peramivir, toremifene citrate salt, and β-d-N4-hydroxycytidine, inhibited SFTSV replication by more than 50% at Cmax, while the other drugs could not inhibit the replication of the SFTSV at Cmax (Figure 1).

### Estimation of the Antiviral Activity of Drugs by Analyzing the Severe Fever With Thrombocytopenia Syndrome Virus Genome Using Reverse Transcription-Polymerase Chain Reaction

**FIGURE 2:**
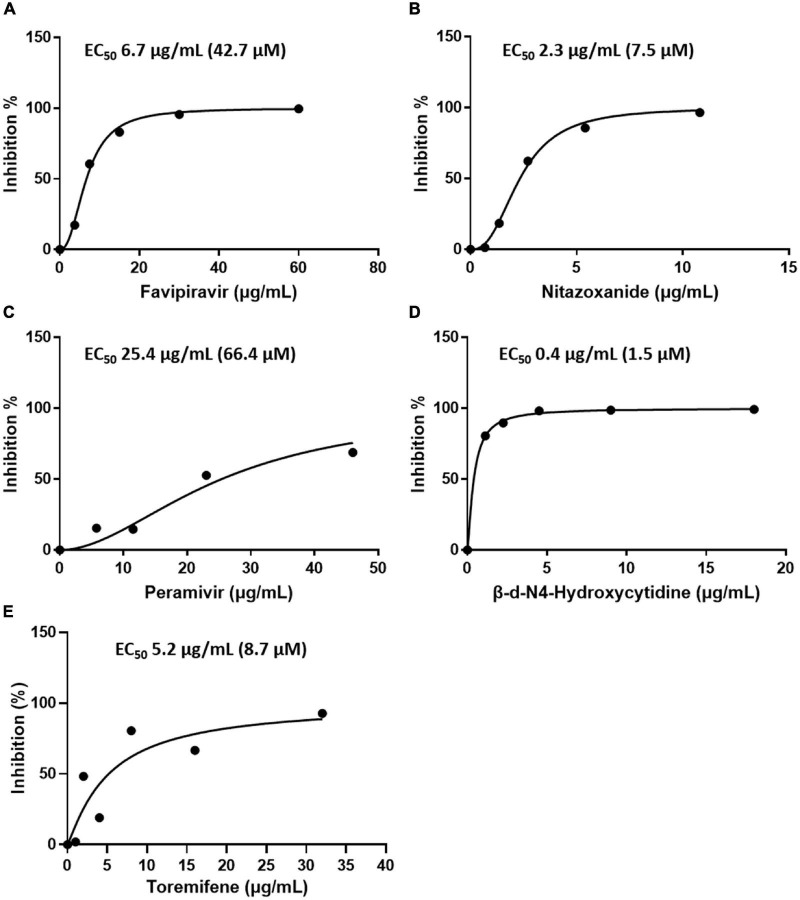
Efficacy of the drugs in inhibiting SFTSV replication in Vero E6 cells as determined by real-time RT-PCR. Graphs depict mean inhibition percentage of SFTSV replication (left y-axis) by antiviral drugs **(A)** favipiravir, **(B)** nitazoxanide, **(C)** peramivir, **(D)** β-d-N4-hydroxycytidine, and **(E)** toremifene. Vero E6 cells were infected with 5 × 10^5^ RNA copies of SFTSV in the presence of a drug for 48 h, after which replication was determined through quantitation of the S segment of the SFTSV genome.

Subsequently, the antiviral activity of the five drugs was determined by comparative analysis of the genome of the SFTSV after treatment with the drugs (concentration: 2-fold serial dilution of Cmax). The concentration of the drugs at which 50% inhibition of viral replication was achieved was calculated in comparison to when no drug treatment was given. The results were as follows: favipiravir, 50% effective concentration (EC_50_) 6.7 μg/mL (42.7 μM); nitazoxanide, EC_50_ 2.3 μg/mL (7.5 μM); peramivir, EC_50_ 25.4 μg/mL (66.4 μM); toremifene citrate salt, EC_50_ > 0.7 μg/mL; and β-d-N4-hydroxycytidine, EC_50_ 0.4 μg/mL (1.5 μM). Favipiravir, nitazoxanide, and peramivir were found to have inhibitory effects on the SFTSV at concentrations below Cmax, and toremifene had inhibitory effects at concentrations higher than Cmax. β-d-N4-hydroxycytidine was not comparable owing to a lack of clinical data (Figure 2 and Table 1).

**TABLE 1:** Efficacy of favipiravir, peramivir, and nitazoxanide against SFTSV replication as determined by real-time RT-PCR and plaque assay.

Drug	EC_50_ (μg/mL) as per RT-PCR	EC_50_ (μg/mL) as per plaque assay	Maximum plasma concentration (Cmax) (μg/mL)
Favipiravir	6.7	4.14	59
Peramivir	25.4	12.9	46
Nitazoxanide	2.3	0.57	10
Toremifene	5.2	1.1	0.7
β-d-N4-hydroxycytidine	0.4	NA^a^	(No clinical data)

*^a^Not available.*

### Antiviral Activity by Plaque Assay

**FIGURE 3:**
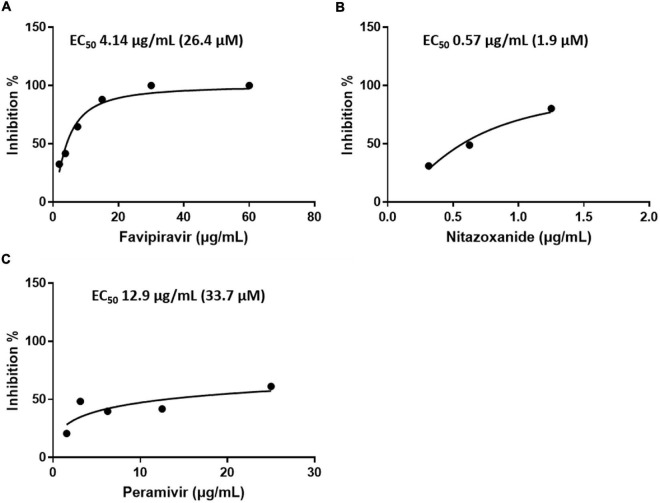
Efficacy of the drugs in inhibiting SFTSV replication in Vero E6 cells as determined by a plaque assay. Graphs depict mean inhibition percentage of SFTS viral plaques (left y-axis) in the presence of the antiviral drugs **(A)** favipiravir, **(B)** nitazoxanide, or **(C)** peramivir. Vero E6 cells were infected with 50–100 PFUs of SFTSV in the presence of a drug for 10 days.

In addition, the antiviral activity of favipiravir, nitazoxanide, and peramivir was determined using plaque assay. The concentrations that inhibited SFTSV by 50% were as follows: favipiravir, EC_50_ 4.14 μg/mL (26.4 μM); nitazoxanide, EC_50_ 0.57 μg/mL (1.9 μM); and peramivir, EC_50_ 12.9 μg/mL (33.7 μM) (Figure 3 and Table 1).

## Discussion

The plasma Cmax and the integrated area under the plasma concentration–time curve (AUC) are considered key pharmacokinetic parameters that enable the translation of clinical drug exposure to a non-clinical study application (14). The plasma Cmax provides an indication of the highest concentration that the subject is exposed to during therapy, and Cmax may be considered an upper limit for drug concentration during *in vitro* studies or the highest plasma exposure for *in vivo* studies to minimize off-target effects (14). Therefore, we infected Vero E6 cells with the SFTSV and cultured them for 48 h in a medium containing Cmax of each drug, and the genome of the SFTSV was quantified in the supernatant to confirm whether viral replication was inhibited. Favipiravir, nitazoxanide, and peramivir inhibited SFTSV by 50% at concentrations lower than Cmax, whereas the toremifene EC_50_ was higher than its Cmax; the results for β-d-N4-hydroxycytidine were not comparable owing to a lack of clinical data.

Studies investigating potential drugs for SFTS-specific therapy have been conducted using existing or newly discovered agents, both *in vitro* and *in vivo* (9). Favipiravir acts by inhibiting viral RNA synthesis; thus, viral RNA is not produced in the infected Vero E6 cells upon drug treatment (15). Favipiravir has demonstrated antiviral activities against all subtypes of influenza virus strains, including type A, B, and C, in studies using laboratory strains of influenza virus, where EC_50_ values of favipiravir ranged from 0.014 to 0.55 μg/L (16). In particular, the EC_50_ of favipiravir in Vero cells against SFTSV was 25 μM, which was lower than that of ribavirin (40 μM) (12). In this study, the EC_50_ 26.4 μM of favipiravir was similar to that found in the previous study. While studying the SFTSV genome by extracting RNA from the cells that were treated with favipiravir, an increase in viral mutation rates and a reduction of intracellular viral RNA levels were reported (17). Hence, there is a possibility that mutations in the SFTSV genome during treatment with the drugs would have blocked the entry of the virus into the cell. However, favipiravir carries a risk for teratogenicity and embryotoxicity (18).

Several studies have reported clinical efficacy of favipiravir in SFTS patients. Favipiravir treatment has been reported to reduce case fatality rate (CFR) (17, 19, 20). In a single-blind, randomized, and controlled trial to assess the efficacy and safety of favipiravir in treating SFTS (ChiCTR1900023350), the favipiravir-treated group showed shorter viral clearance, lower incidence of hemorrhagic signs, and faster recovery of laboratory abnormities than the controls (17).

Nitazoxanide was originally developed and commercialized as an antiprotozoal agent and was later identified as a first-in-class broad-spectrum antiviral agent that then got repurposed for the treatment of influenza (21). Studies pertaining to the mechanism of action of nitazoxanide against influenza viruses have shown that the drug blocks maturation of viral hemagglutinin at the post-translational stage (22). The drug had no effect on other glycoproteins, neuraminidase, the target of oseltamivir and zanamivir, or the M2 protein, the target of amantadine, and it had no effect on viral infectivity, adsorption, or the entry of the virus into target cells (23, 24). A phase 2b/3 clinical trial found that the oral administration of nitazoxanide (600 mg twice daily for 5 days) reduced the duration of clinical symptoms as well as the viral shedding compared to that observed for placebo administered individuals with laboratory-confirmed influenza (25). Tizoxanide, the active circulating metabolite of nitazoxanide, inhibited the replication of influenza A/H1N1 *in vitro* at an EC_50_ of 0.3 μg/mL (22). Additionally, nitazoxanide inhibited SARS-CoV-2 in Vero E6 cells at a low micromolar concentration with an EC_50_ of 2.12 μM (26). Another study reported that nitazoxanide was able to inhibit the replication of Japanese encephalitis virus (JEV) in cultured cells and reduced the mortality of mice challenged with a lethal dose of JEV (23). In case of hepatitis C virus (HCV) infection, nitazoxanide increased eukaryotic initiation factor 2α phosphorylation, a modification known to mediate host antiviral defenses (27). In our study, we found that nitazoxanide at a concentration lower than Cmax inhibits SFTSV replication, however, its exact mechanism needs to be explored in the future.

Peramivir is an antiviral agent that blocks viral growth by selectively inhibiting neuraminidase (NA), an enzyme that releases virus particles from infected cells during human influenza A and B infections, and is administered once daily through an intravenous route (28). NA inhibitors (NAIs), zanamivir, oseltamivir, peramivir, and laninamivir, inhibit the NA activity of the viral particles at the surfaces of the infected cells, thereby reducing the release of the viral particles from the cell surface to other cells for the next round of infection. NAIs cause the accumulation of virus particles on cell surfaces and inhibit the spread of viral infection (15). There is no report that peramivir has any antiviral activity other than as an NAI.

In the present study, the anti-influenza drugs peramivir and nitazoxanide inhibited SFTSV replication at a concentration below Cmax in Vero E6 cells. However, other anti-influenza drugs, such as baloxavir marboxil and oseltamivir, had no effect. Peramivir is an approved antiviral drug that can be prescribed to patients with influenza in South Korea (29). In addition, it is essential to study the mechanisms that can demonstrate the effect of peramivir against SFTSV.

Our study has the following limitations: 1) We did not confirm the efficacy of the tested drugs in other SFTSV genotypes; however, the effect of the drug may depend on the genotype of the virus. 2) We only performed *in vitro* experiments and did not validate their results with *in vivo* experiments. 3) This study does not comment upon the mechanism of action of the identified therapeutic candidates for SFTS.

In conclusion, on exploring the effects of antiviral drugs against the SFTSV using an *in vitro* antiviral activity assay, we found favipiravir, peramivir, and nitazoxanide to exert potential inhibitory effects at concentrations below Cmax. To the best of our knowledge, there have been no previous studies that report that nitazoxanide and peramivir inhibit the replication of SFTSV. Therefore, this study suggests the possibility of using nitazoxanide and peramivir for the treatment of SFTS. Future studies on these drugs should include animal models of SFTSV infection, clinical trials including dose-ranging trials, and evaluation of combination therapy with other potential antivirals against SFTSV.

## Data Availability Statement

The original contributions presented in the study are included in the article. Further inquiries can be directed to the corresponding author.

## Author Contributions

M-SB performed the investigation and drafted the manuscript. C-MK prepared the methodology and performed the analysis. D-MK conceived the study and revised the manuscript. NY supervised the study. All authors contributed to the article and approved the submitted version.

## Conflict of Interest

The authors declare that the research was conducted in the absence of any commercial or financial relationships that could be construed as a potential conflict of interest.

## Publisher’s Note

All claims expressed in this article are solely those of the authors and do not necessarily represent those of their affiliated organizations, or those of the publisher, the editors and the reviewers. Any product that may be evaluated in this article, or claim that may be made by its manufacturer, is not guaranteed or endorsed by the publisher.
